# Chronic widespread musculoskeletal pain shares a highly heritable latent pathway with atherosclerosis and arterial stiffness

**DOI:** 10.1097/j.pain.0000000000003486

**Published:** 2024-12-03

**Authors:** Maryam Kazemi Naeini, Marina Cecelja, Maxim B. Freidin, Isabelle Granville Smith, Pirro Hysi, Christopher Sivert Nielsen, Frances M. K. Williams

**Affiliations:** a Department of Twin Research and Genetic Epidemiology, School of Life Course and Population Sciences, King's College London, London, United Kingdom; b Department of Biology, School of Biological and Behavioural Sciences, Queen Mary University of London, London, United Kingdom; c Division of Mental and Physical Health, Norwegian Institute of Public Health, Oslo, Norway; d Division of Emergencies and Critical Care, Department of Pain Management and Research, Oslo University Hospital, Oslo, Norway

**Keywords:** Chronic widespread pain, Arterial stiffness, Atherosclerosis, Carotid–femoral pulse wave velocity, Carotid intima–media thickness, Carotid plaque

## Abstract

Chronic widespread pain (CWP) is prevalent and associated with reduced life expectancy. Cardiovascular disease is one possible mechanism for this. The purpose of this study was to examine the association of CWP with arterial stiffness and carotid plaque measured using ultrasound to determine if shared environmental or genetic factors might account for any observed association. Around 3000 participants from the TwinsUK with CWP information and measures of carotid–femoral pulse wave velocity (cfPWV), carotid intima–media thickness (cIMT), and plaque were considered. The relationship between CWP and cfPWV, cIMT, and plaque was determined. UK Biobank data were used to replicate the association. Cholesky decomposition and multivariate pathway twin models were examined. Using a 2-sample Mendelian randomisation approach, the causal association between CWP and coronary artery disease was assessed. TwinsUK participants demonstrated a significant association between CWP and increased cfPWV consistent with arterial stiffening (OR = 1.35, *P*-value = 0.012), as well as the presence of carotid plaque (OR = 1.45, *P*-value = 0.8e-5). The twin modelling showed a common latent component and pathway underlying CWP, cfPWV, and carotid plaque, with genetic factors accounting for 68% and 90% of the latent factor variation, respectively. The 2-sample MR revealed a potential causal association between CWP and coronary artery disease. This study found that those with CWP have increased the risk of arterial stiffness and atherosclerosis and suggests that CWP leads to an increased risk of cardiovascular disease through genetic factors.

## Abstract 2

A common latent factor mediates chronic widespread pain (CWP), arterial stiffness, and atheromatous plaque association, explaining 65% of CWP, 26% of carotid–femoral pulse wave velocity, and 34% of plaque genetic variations.

## 1. Introduction

Chronic widespread musculoskeletal pain (CWP) is a complex condition with uncertain pathophysiology, affecting 5% to 15% of the general population.^30^ Chronic widespread pain heritability estimates based on twin studies vary from 48% to 58%, indicating a significant genetic influence in different populations.^8,21^ Chronic widespread pain heritability based on genetic variants has been estimated at 33% in a northern European population.^37^ Chronic widespread pain is a key diagnostic criterion for fibromyalgia, and CWP imposes a considerable economic burden in terms of disability, work absence, and medical expense.^30^

People with CWP manifest high rates of comorbidity with cardiovascular disease (CVD) along with anxiety, depression, and fatigue.^5-9^ A recent meta-analysis found all-cause and CVD mortality risk ratios in people with CWP of 2.43 and 3.24, respectively.^29^ Twin studies demonstrate a shared heritability between CWP and self-reported CVD as well as fatigue and affective disorders.^7,18,50^ These findings suggest the presence of shared genetic factors, which may be leveraged for prediction, monitoring, and the co-occurrence comorbid diseases. Furthermore, CWP may itself be a neglected CVD risk factor.^2,39,52^ Among older adults in that sample, the experience of chronic pain has been associated with subsequent CVD incidents; reduced physical activity, poor sleep, and diet may mediate the part of this risk.^38^ A Mendelian randomisation (MR) study has suggested a causal relationship between multisite chronic pain and CVD subtypes, even when taking account of mental illness, smoking, physical activity, and body mass index.^27^

Atherosclerosis is the formative, hallmark pathology of CVD. It is a chronic inflammatory disease that changes arterial wall structure with gradual lipid accumulation and growth of atherosclerotic plaque which impairs arterial function.^4^ Globally, atherosclerosis is the primary cause of CVD mortality and stroke.^22,44^ Arterial wall damage attracts macrophages and inflammatory proteins, insufficient clearing of these cells combined with higher levels of low-density lipoprotein (LDL) cholesterol molecules embed in endothelium tissue forming a fibrous cap. More developed atherosclerosis attracts calcium deposits from the blood, further enlarging the plaque.^4^ Atherosclerosis has a complex aetiology that includes interaction between environmental, systemic and genetic factors, and epigenetic regulation.^4^

The severity of atherosclerosis and volume of plaque accumulation may be inferred by measures of carotid intima–media thickness (cIMT) and carotid plaque.^32^ Carotid plaque and cIMT have predictive value for future CVD events, especially in at-risk populations such as those with diabetes; they may provide the best noninvasive single CVD predictive assessment.^10^

Arterial stiffening measured, measured using carotid–femoral pulse wave velocity (cfPWV),^3,23^ is a major predictor of CVD morbidity and mortality events.^25^ Molecular causes of arterial stiffness are localised to the medial part of the arterial wall and involve elastin/collagen changes, inflammation, and advanced glycation end products.^1^

The aim of the present study was to investigate the association of CWP with measures of atherosclerosis (carotid IMT and plaque) and arterial stiffness (measured as cfPWV). We examined the heritability of atherosclerosis, arterial stiffness, and CWP and sought evidence of shared genetic factors contributing to their association.

## 2. Methods

### 2.1. Study participants

Data from the TwinsUK cohort, a nationwide sample of monozygotic (MZ) and dizygotic (DZ) twins, were used. Since 1992, TwinsUK has collected multiple questionnaires, physical and cognitive measurements, and biological data from over 15,000 adult twin participants, male and female, aged older than 18 years.^51^ The majority of whom are female (88%) based on the original registry funding and setup. The registry was established in 1992 with the primary goal of investigating osteoporosis and osteoarthritis. As such conditions are common in women, several hundred middle-aged women were recruited to form the basis of the first register.

Participants from TwinsUK were eligible for this study if they had completed pain questionnaires and undergone cIMT and cfPWV measurements during clinical visits between 2006 and 2016. Twin zygosity was validated by DNA genotyping. The participants provided informed consent, and the ethics committee had granted the approval.

UK Biobank (UKB) recruited half a million 40–69-year-old participants living in England, Wales, and Scotland between 2006 and 2010. The assessment visit underwent additional enhancements as recruitment was completed, with significant groups of the cohort having a series of ultrasound examinations in the imaging visit from 2014 and a pulse wave velocity measure in the initial assessment visit between 2006 and thereafter until 2017.^45^

This study is a cross-sectional study from TwinsUK to investigate the effect of CWP on cIMT, cfPWV, and carotid plaque. We used the UKB study to replicate the cross-sectional effect of CWP findings. The aims of the study were to (1) investigate the epidemiological connection of CWP with arterial stiffness (cfPWV) and atherosclerosis (cIMT, carotid plaque) using TwinsUK data and replicate the findings in UKB data, (2) using twin modelling to evaluate the direct and indirect impacts of genetic and environmental variables on this connection, and (3) investigating the causal relationship between CWP and coronary artery disease.

### 2.2. Assessment instruments

#### 2.2.1. Chronic widespread musculoskeletal pain

CWP was identified using the self-reported London Fibromyalgia Epidemiology Symptom Screening questionnaire in TwinsUK.^54^ CWP was defined as musculoskeletal pain lasting 3 months or longer in the left and right sides of the body, above and below the diaphragm; and those reporting a doctor's diagnosis of fibromyalgia.^55^ Twins who were pain free or had pain less than 3 months or pain that did not meet criteria were considered controls in this study.

UK Biobank defined CWP cases based on self-reported questionnaire, which included pain all over the body that persists for more than 3 months as the diagnosis (field ID 6159). Those who reported no pain in the last month, pain all over the body lasting less than 3 months, or reported nonmusculoskeletal pain (headache, facial, and abdominal pain) were considered controls.

#### 2.2.2. Pulse wave velocity

In TwinsUK, measurements were performed in a vascular laboratory with air temperature controlled at 22 to 24°C. After participants had rested at least 10 minutes, brachial blood pressure was measured 3 times using a certified oscilloscope (Omron 705CP, Omron, Tokyo, Japan). The SphygmoCor system (Atcor Medical, Sydney, Australia) was used for cfPWV measurements where the carotid and femoral pulse were recorded using applanation tonometry referenced to ECG. The path distance between the carotid and femoral sites was measured by the distance at the legend point between the femoral artery and the sternal notch, as previously described,^11^ in line with current guidelines.^5^

In UKB, the pulse wave as an arterial stiffness index (field ID 21021) was measured using the finger volume waveform. This index is directly related to the time it takes for the pulse wave to pass through the arterial tree in the lower body and reflect to the finger. The stiffness index is determined by dividing the peak-to-peak time by the height of the person.^48^ The normal cutoff value for PWV was established at 10 m/second using European Society of Cardiology/European Society of Hypertension recommendations. Values higher than this were considered abnormal.^5^

#### 2.2.3. Carotid intima–media thickness and carotid plaque

In TwinsUK, a 13 MHz vascular probe and a Siemens CV70 (Siemens, Erlangen, Germany) B-mode ultrasound machine were used to examine the left and right carotid and femoral arteries. Using automated wall tracking software (Medical Imaging Applications, Coralville, Iowa), normal carotid IMT was measured in the distal and proximal walls, 1 to 2 cm proximal to the carotid bifurcation, during diastole in a region devoid of apparent plaque. Mean IMT values in near and distant walls were used, as previously.^11^ The American Society of Echocardiography recommends using cIMT values ≥75th percentile to classify cIMT as abnormal.

The common carotid, carotid bifurcation, and femoral artery were inspected for plaque on arterial walls. Plaque was identified in the longitudinal view as localised expansion and protrusion into the lumen with a thickness of ≥1.5 mm relative to neighbouring regions and verified in transverse view.^11^ Measurements were taken by skilled observers who were not aware of the participants' clinical information.

In UKB, cIMT measurements were collected during imaging visit assessments in 2014. The 2D ultrasound scan measures cIMT at 4 predetermined angles for each carotid, giving the sonographer real-time angle measurements. The distal common carotid was scanned along the long axis, and the flow divider between the exterior and internal carotid arteries was identified. The average of 4 mean cIMT values, 2 for each of the left and right carotid arteries, was applied as the cIMT mean for the final phenotype (field ID 22671-22680).

### 2.3. Statistical analyses

#### 2.3.1. Association analysis

Descriptive statistics including the frequency and percentage for categorical variables, and median and interquartile ranges, were reported based on non-normally distributed continuous variables. The generalized estimating equation (GEE) logistic regression modelling was adjusted for sex, age, body mass index (BMI), mean arterial pressure, and twin status. In UKB, linear regression investigated the association between CWP and atherosclerosis biomarkers. Generalized estimating equation model is designed to manage correlated data by estimating the parameters of a generalised linear model while accounting for correlation within clusters. In twin studies, each twin pair is treated as a cluster. The twins' responses are considered related. Generalized estimating equation estimates the average response across clusters (twin pairs), accounting for within-pair correlation.^16^

Based on the strong linear correlation between atherosclerosis biomarkers and age and sex, subgroup analyses were conducted. Individuals aged older than 50 years had considerably greater PWV and cIMT than younger individuals; therefore, the subgroup analyses were conducted in younger and older male participants and female participants with the age cut point at 50 years.

**Figure 1.**
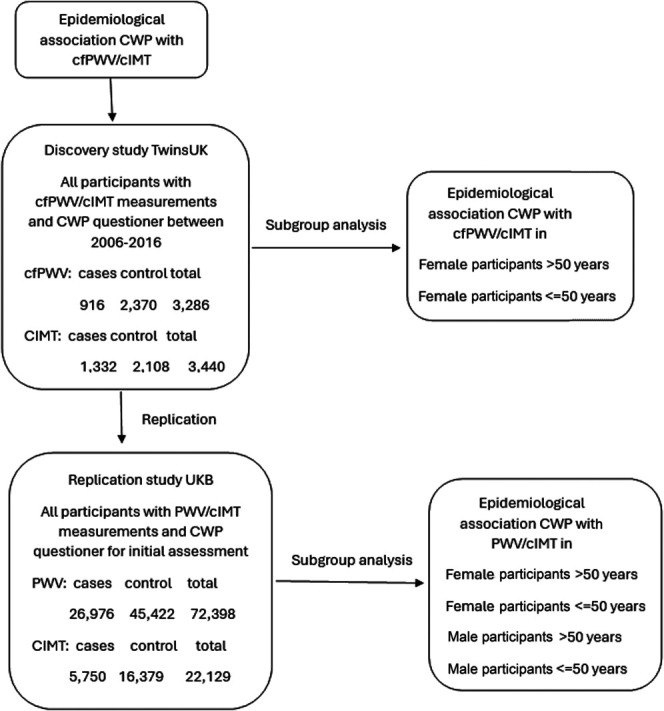
Association analysis of CWP with cIMT and cfPWV flowchart in TwinsUK and UKB. cfPWV, carotid–femoral pulse wave velocity; cIMT, carotid intima–media thickness; CWP, chronic widespread pain; UKB, UK Biobank.

We used UKB data set to examine the association between CWP with cIMT and pulse wave velocity as an arterial stiffness index in the whole sample by CWP status and the subgroups, as above (male and female, above and below the age of 50 years). The *gee* and *stats* packages in R were used, and all statistical tests were conducted using a 5% significance threshold. The sensitivity analysis was conducted in the final stage by re-running the models with smoking status as an additional covariate. Figure 1 shows the association analysis process flowchart.

#### 2.3.2. Shared heritability and twin model analysis

Structural equation twin modelling was performed using the maximum likelihood approach (UMx package in R^8^), and the parameters of the full Cholesky model and its submodels were estimated, along with the related bootstrap confidence intervals. Models were based on twin phenotypic variance and covariance, which can be partitioned into additive genetic (A), shared environmental factors (C), and unique environmental factors (E). Likelihood ratio tests were used to assess the significance of each parameter, and most favourable model was selected based on the optimal balance between goodness of fit and parsimony using the Akaike information criterion (AIC). Alternative multivariate pathway models, including common pathway, were examined to investigate if a common factor could explain the covariation between phenotypes.

#### 2.3.3. Two-sample Mendelian randomization

We conducted bidirectional, 2-sample MR to investigate the causal association between CWP and coronary artery disease. For CWP, we used the summary statistics of GWAS using UKB data with 249843 samples, and for coronary artery disease GWAS summary statistics, we used a large-scale GWAS meta-analysis released by the CARDIoGRAMplusC4D study, with 60,801/123,504 samples. Most of the participants included had European ancestry.^33^

For selecting instrumental variables, we used independent single-nucleotide polymorphisms (SNPs) that were significantly linked with the exposure and outcome at the genome-wide level (*P* < 5 × 10^−8^). We also estimated the F statistics to assess the strength of genetic instruments, using F statistics greater than 10 to prevent weak instrument bias. Data harmonisation was performed to verify that SNP alleles were aligned for both exposure and outcome. We used a clumping technique with R2 = 0.001 and a 10,000 kb window size to exclude the SNP associated with significant linkage disequilibrium. Considering CWP as exposure, after clumping, just 3 variants were retained as IVs, which were the same reported hits in the CWP GWAS.^37^ 29 SNPs were selected as IVs for the coronary artery disease exposure. To integrate the effect (Wald ratio) of each SNP, we used the inverse-variance weighted (IVW) approach as the primary analysis. Although the IVW approach assumes that all genetic instruments are legitimate, imbalanced horizontal pleiotropy may skew its causal estimations. So, we additionally conducted pleiotropy-robust MR sensitivity studies using the weighted median. An online calculator was used to determine poststatistical power for 2-sample MR (https://shiny.cnsgenomics.com/mRnd/).^6^ The MR and ieugwasr R packages were used for these analyses.^56^

## 3. Results

### 3.1. Chronic widespread pain and arterial stiffness association (carotid–femoral pulse wave velocity)

TwinsUK sample characteristics are presented in Table 1. Of the 3286 TwinsUK participants, the majority were female (92.3%), the median age was 59 years (IQR 51-67 years), the median BMI was 25.36 kg/m^2^ (IQR 22.45-28.27), and the median arterial pressure (MAP) was 92 mm Hg (IRQ: 84-100). The median cfPWV in TwinsUK was 8.8 m/second (IRQ 7.8-10.2). Of the 916 individuals considered in high-risk group of cfPWV, 36.9% reported CWP, whereas among the 2370 participants with a normal cfPWV measure, 24.9% had CWP. The association of CWP with cfPWV was significant using a GEE logistic regression model adjusting for age, sex, BMI, MAP, and twin relationship, (OR = 1.35 (1.06-1.71), *P*-value = 0.012) (Table 2). In the next step, due to the small number of male participants (n = 253) in the TwinsUK sample, further regression analyses were conducted in female participants only (Table 3). The association between CWP and cfPWV was significant after adjusting for age and BMI (OR = 1.41 (1.09-1.81), *P*-value = 0.007) in female participants aged older than 50 years. Having CWP increased the odds of being in the at-risk group for cfPWV and arterial stiffness.

**Table 1:** Baseline characteristics of TwinsUK participants.

Variables	Sample size	Descriptive statistics
Sex (female)	3286	3033 (92.3%)
Age (y)	3286	59 (IRQ: 50-66)
BMI (kg/m^2^)	3286	25.36 (IRQ: 22.81-28.63)
CWP	3286	927 (28.2%)
cIMT (mm)	3440	0.66 (IRQ: 0.59-0.74)
cfPWV (m/s)	3286	8.80 (IRQ: 7.80-10.20)
cIMT (at risk)*	3440	1332 (38.7%)
cfPWV (at risk)†	3286	916 (27.9%)
Carotid plaque	3508	967 (27.6%)
MAP (mm Hg)	3367	92 (IRQ: 84-100)

* cIMT > 75th percentile.

† cfPWV > 10 (m/s).

BMI, body mass index; cfPWV, carotid–femoral pulse wave velocity; cIMT, carotid intima–media thickness; CWP, chronic widespread pain; IQR, interquartile range; MAP, mean arterial pressure.

**Table 2:** Association between chronic widespread pain with atherosclerosis and arterial stiffness in TwinsUK.

Variables	cIMT (n = 2736)			Plaque (n = 3485)			cfPWV (n = 3302)		
	OR	CI	*P*	OR	CI	*P*	OR	CI	*P*
Sex (male)	1.70	1.19-2.44	0.003	2.31	1.68-3.16	1.7e-7	1.22	0.83-1.77	0.302
Age	3.20	2.80-3.65	0.1e-35	3.95	3.48-4.48	0.1e-40	6.05	4.96-7.38	0.1e-40
BMI	1.00	0.95-1.05	0.022	1.10	1.00-1.21	0.036	1.31	1.17-1.46	2.9e-6
CWP	1.25	1.00-1.55	0.042	1.45	1.20-1.75	8.5e-4	1.35	1.06-1.71	0.012
MAP	—	—	—	—	—	—	2.98	2.57-3.46	0.1e-30

Standardised scores are considered in model for age, MAP, and BMI.

BMI, body mass index; cfPWV, carotid–femoral pulse wave velocity; CI, confidence interval; cIMT, carotid intima–media thickness; CWP, chronic widespread pain; MAP, mean arterial pressure; OR, odds ratio.

**Table 3:** Predictors of carotid–femoral pulse wave velocity in TwinsUK, by subgroup.

(cfPWV)	Female participants younger than 50 (n = 792)			Female participants older than 50 (n = 2241)		
	OR	CI	*P*	OR	CI	*P*
Age	1.37	0.22-8.29	0.730	6.67	5.26-8.45	0.1e-40
BMI	1.74	1.11-2.73	0.015	1.25	1.11-1.42	0.2e-4
CWP	0.46	0.10-2.15	0.330	1.41	1.09-1.81	0.007
MAP	4.40	2.44-7.94	7.2e-7	2.82	2.41-3.31	0.1e-30

Multivariable regression analysis between cfPWV and CWP.

Standardised scores are considered in model for age, MAP, and BMI.

BMI, body mass index; cfPWV, carotid–femoral pulse wave velocity; CI, confidence interval; CWP, chronic widespread pain; MAP, mean arterial pressure; OR, odds ratio.

UK Biobank sample characteristics are presented in Table 4. Using data from 72,373 UKB participants with PWV and reported CWP status, a significant association between PWV index and CWP (β = 0.28 (0.15-0.47), *P*-value = 1.4e-4) was found (Table 5). Subgroup analysis showed that the increasing mean PWV index in female participants aged older than 50 years was related to CWP (β = 0.27 (0.07-0.47), *P*-value = 0.006). The association was also significant in male participants younger than 50 years (β = 0.86 (0.51-4.61), *P*-value = 0.3e-10) (Table 6).

**Table 4:** Baseline characteristics of participants in the UK Biobank study.

Variables	Sample size	Descriptive statistics
Sex (% female)	72,398	37,604 (52.0%)
Age (y)	72,398	59 (IRQ: 51-64)
BMI (kg/m^2^)	72,398	26.36 (IRQ: 23.88-29.30)
CWP	72,398	2106 (2.9%)
cIMT (mm)	22,129	0.67 (IRQ: 0.60-0.76)
PWV (m/s)	72,398	8.89 (IRQ: 6.80-11.08)
cIMT (at risk)*	22,129	5750 (26.0%)
PWV (at risk)†	72,398	26,976 (37.3%)
Carotid plaque	—	—

* cIMT > 75th percentile.

† cfPWV > 10 (m/s).

BMI, body mass index; cIMT, carotid intima–media thickness; CWP, chronic widespread pain; IQR, interquartile range; PWV, pulse wave velocity (arterial stiffness index).

**Table 5:** Association between chronic widespread pain with carotid intima–media thickness and carotid–femoral pulse wave velocity in UK Biobank.

Variables	cIMT (n = 22,129)			PWV (n = 72,373)		
	Beta	CI	*P*	Beta	CI	*P*
Sex (male)	0.035	0.032-0.038	5.1e-40	1.09	1.05-1.14	1.7e-10
Age	0.054	0.052-0.056	0.1e-40	0.63	0.61-0.65	1.5e-10
BMI	0.010	0.008-0.011	1.1e-33	0.30	0.28-0.32	2.5e-5
CWP	0.005	−0.003-0.018	0.440	0.28	0.15-0.47	1.4e-4

Standardised scores are considered in model for age and BMI.

Beta, Regression coefficient; BMI, body mass index; CI, confidence interval; cIMT, carotid intima–media thickness; CWP, chronic widespread pain; PWV, pulse wave velocity (arterial stiffness index).

**Table 6:** Multivariable regression analysis between chronic widespread pain and carotid–femoral pulse wave velocity: UK Biobank by subgroup.

(PWV)	Male participants younger than 50 (n = 8220)			Male participants older than 50 (n = 26,567)		
	Beta	CI	*P*	Beta	CI	*P*
Age	0.94	0.80-1.08	1.01e-41	039	0.33-0.45	2.23e-43
BMI	0.37	0.31-0.43	1.09e-32	0.30	0.26-0.35	4.49e-46
CWP	0.86	0.50-1.23	0.3e-10	0.15	−0.09-0.39	0.21

(PWV)	Female participants younger than 50 (n = 9123)			Female participants older than 50 (n = 28,488)		
	Beta	CI	*P*	Beta	CI	*P*
Age	0.69	0.57-0.82	1.8e-27	0.50	0.44-0.56	1.1e-63
BMI	0.24	0.19-0.28	1.9e-25	0.30	0.26-0.34	1.8e-56
CWP	0.17	−0.08-0.44	0.189	0.27	0.07-0.47	0.006

Standardised scores are considered in model for age and BMI.

Beta, Regression coefficient; BMI, body mass index; cfPWV, carotid–femoral pulse wave velocity; CI, confidence interval; cIMT, carotid intima–media thickness; CWP, chronic widespread pain; PWV, pulse wave velocity (arterial stiffness index).

### 3.2. Chronic widespread pain and atherosclerosis biomarkers association (carotid intima–media thickness and plaque)

Of 2736 twin participants having both cIMT measurement and CWP status, 90.2% were female with the median age of 59 years (IRQ 50-67 years) and median BMI of 25.44 (IRQ 22.80-28.77). The frequency of CWP in the normal cIMT group (n = 1633) was 23.5%, and in the at-risk group of cIMT (n = 1103), it was 33.8%. Presumably, this is a positive association for unadjusted association. In multivariable analysis using the GEE logistic regression model, after adjustment for sex, age, BMI, and twin pair correlation, the CWP association was significant for increasing odds of at-risk cIMT (OR = 1.25, *P*-value = 0.04) (Table 2). After excluding male participants and separating female participants into above and below 50 years subgroups, there was no significant association.

Using UKB data in 22,129 participants with cIMT and CWP information (Table 5), the association between cIMT outcome and CWP using linear regression model with adjusting for sex, age, and BMI was not significant (*P*-value = 0.357) and subgroup analysis was also not significant. There was a significant association between CWP and plaque as an outcome, in 3458 TwinsUK participants using GEE logistic regression model with adjustment for sex, age, BMI, and twin relationship (OR = 1.45 (1.20-1.75), *P*-value = 8.5e-4) (Table 2). In female participants older than 50 years, the CWP can increase the odds of carotid plaque presence (OR = 1.51, *P*-value = 4.5e-4).

### 3.3. Shared heritability between carotid–femoral pulse wave velocity and chronic widespread pain (twin model analysis)

**Figure 2.**
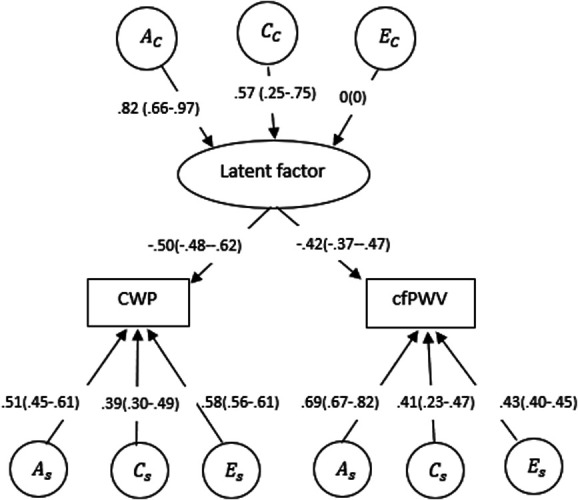
The standardised path coefficients in the AC common pathway between CWP and cfPWV are derived from latent genetic and environmental factors. Ac, Cc, and Ec denote common additive genetic, shared environmental, and unique environmental effects. (A, C, and E) denoted trait-specific additive genetic, shared environmental, and unique environmental effects, respectively. Squares of the path coefficients give corresponding variance proportions. cfPWV, carotid–femoral pulse wave velocity; CWP, chronic widespread pain.

We performed twin modelling using 945 MZ and 807 DZ twins to ascertain the genetic relationship of both dichotomous cfPWV and CWP. Using Mx package, the 3 types of correlation were assessed. First, a cross-trait correlation of 0.23 was estimated as a phenotypic correlation. The second was a within-pair correlation of 0.69 for cfPWV and 0.54 for CWP, and the cross-twin cross-trait correlation was 0.20. The fitting of the multivariate Cholesky decomposition model revealed that the ACE model with the lowest AIC was the best fitting Cholesky model (Table 7), indicating interrelation between the cfPWV and CWP. The parameter estimates revealed that 7% (0.26^2^ (bootstrap CI: 0.17-0.36)) of the variance was explained by genetic effects that are shared between CWP and cfPWV. The best common pathway model was the AC model (Table 8), and its corresponding estimates are displayed in Figure 2. The Common Pathway concept demonstrates common ($A_{C}$, $C_{C}$, $E_{C}$) and specific ($A_{s}$, $C_{s}$, $E_{s}$) genetic and environmental effects on the CWP and cfPWV. The latent factor's paths to the CWP and cfPWV show how much variance it explains in each phenotype. The observed covariation pattern between CWP and cfPWV was due to a latent common pathway, of which $A_{C}$ = 68% (0.82^2^; squares of the path coefficients give corresponding variance proportion) was explained by genetic factors and $C_{C}$ = 32% (0.57^2^) by common environmental ones.

**Table 7:** Standardized path loadings and goodness-of-fit statistics for multivariate Cholesky models of chronic widespread pain and atheroma biomarkers.

CWP and cfPWV						
Cholesky models	A	C	E	−2LL	Df	AIC
ACE	**0.26**	**0.11**	**−0.03**	**7206.715**	**6535**	**7228.71**
CE	—	0.26	0.03	7245.16	6532	7261.16
AE	0.26	—	−0.03	7214.62	6532	7230.62
E	—	—	0.20	7727.04	6529	7737.04

CWP and plaque						
Cholesky models	A	C	E	−2LL	Df	AIC
ACE	**−0.02**	**0.46**	**0.10**	**6581.487**	**6003**	**6603.49**
CE	—	0.31	0.08	6604.397	6000	6620.40
AE	0.28	—	0.07	6596.876	6000	6612.18
E	—	—	0.27	7033.961	5997	7043.92

Most suitable models are shown bold.

−2LL, −2 Log likelihood; AIC, Akaike information criterion; cfPWV, carotid–femoral pulse wave velocity; CWP, chronic widespread pain; df, Degree of freedom.

**Table 8:** CP model common factor path loadings and goodness-of-fit twin models of CWP and atheroma biomarkers.

CWP and cfPWV						
Common pathway model	AC	CC	EC	−2LL	Df	AIC
ACE	0.82	0.57	0.0	7206.965	6535	7232.965
AC	**0.82**	**0.56**	**-**	**7206.965**	**6534**	**7230.965**
AE	1.0	—	0.0	7207.734	6534	7231.734
E	—	—	0.60	7240.842	6533	7262.842

CWP and plaque						
Common pathway model	AC	CC	EC	−2LL	Df	AIC
ACE	0.95	0.0	0.30	6588.105	6002	6614.105
AE	**0.95**	**-**	**0.30**	**6588.105**	**6001**	**6612.65**
AC	1.0	0.0	—	6588.715	6001	6612.715
E	—	—	1.0	6621.089	6000	6643.089

Most suitable models are shown bold.

−2LL, −2 Log likelihood; AIC, Akaike information criterion; cfPWV, carotid–femoral pulse wave velocity; CP, common pathway model; CWP, chronic widespread pain; df, Degree of freedom.

Figure 2 shows that specific genetics accounts for $A_{s}$ = 0.51^2^ ∼ 26% of the variation in CWP. However, common genetics through the latent component would explain 0.82^2^ × 0.50^2^ ∼ 17% of the variation in CWP. The study suggests that the latent component accounts for 0.17/0.26 ∼ 65% of the genetic variation in CWP. In cfPWV, specific genetics account for $A_{s}$ = 0.69^2^ ∼ 47% of the variance in cfPWV. However, common genetics through the latent component might explain 0.82^2^ × 0.42^2^ = ∼12% of the variance in cfPWV. The analysis indicates that the latent component accounts 0.12/0.47 ∼ 26% of the genetic variance in cfPWV.

**Figure 3.**
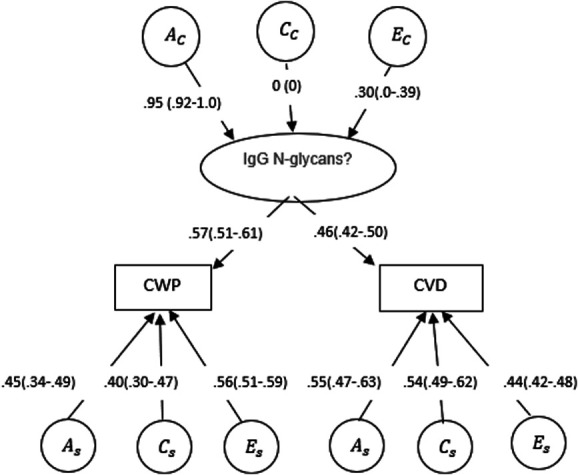
The standardised path coefficients in the AE common pathway between CWP and carotid plaque derived from latent genetic and environmental factors. Ac, Cc, and Ec denote common additive genetic, shared environmental, and unique environmental effects. (A, C, and E) denoted trait-specific additive genetic, shared environmental, and unique environmental effects, respectively. Squares of the path coefficients give corresponding variance proportions. CWP, chronic widespread pain.

The same twin common pathway model was fitted to 763 DZ and 853 MZ twin pairs to investigate the potential common pathway behind carotid plaque occurrence in people with CWP. Figure 3 shows the results of the AE common pathway model (Table 8), and it shows that there is a common pathway that can be explained $A_{C}$ = 90% (0.95^2^) by genetic components and $E_{C}=$ 9% (0.30^2^) by unique environmental factors. Figure 3 reveals that specific genetics account for $A_{s}$ = 0.45^2^ ∼ 20% of the variance in CWP. However, common genetics through the latent component might explain around 30% of the variance in CWP (0.95^2^ × 0.57^2^). The analysis indicates that the latent component explains for 0.20/0.30 ∼ 67% of the genetic variance in CWP. Specific genetics explain approximately $A_{s}$ = 30% of the diversity in carotid plaque. However, common genetics through the latent component may explain approximately 19% of the variation in carotid plaque (0.95^2^ × 0.46^2^). The research shows that the latent component explains for 0.19/0.55 ∼ 34% of the genetic variation in carotid plaque. The small difference between CWP $A_{s}$ in these 2 models can be attributed to the fact that we used 2 separate data sets for analysis: CWP and cfPWV and CWP and carotid plaque, and samples are not totally overlapped.

### 3.4. Casual association between chronic widespread pain and risk of coronary artery disease

As presented in Table 9, genetic liability for CWP showed a strong association with a higher risk of coronary artery disease (IVW method: OR = 7.24, *P*-value = 0.001) using 3 independent GWAS significant variants, which were the same variants reported in our GWAS (rs10490825, rs1491985, and rs165599) with F-statistic equal 31.7, but the reverse direction did not support a significant causal effect of coronary artery disease on CWP (*P*-value = 0.368). The post hoc power calculation for the MR result showed 100% power with the sample sizes for the estimated OR.

**Table 9:** Causal effect of chronic widespread pain on coronary artery disease in 2-sample MR.

Method	OR	SE	CI	*P*
Weighted median	7.17	2.14	1.52-30.26	0.012
Inverse variance weighted	7.24	1.84	2.17-24.04	0.001
Robust IVW	7.20	1.47	3.36-15.44	0.000

CI, confidence interval of OR; IVW, inverse variance weighted; OR, odds ratio.

## 4. Discussion

Chronic widespread pain is a risk factor of premature death from cardiovascular disease in epidemiological studies.^29^ Our findings suggest that there is an association between CWP and measures of atherosclerosis (carotid IMT and plaque) and aortic stiffness (cfPWV). We report a weak association between CWP and cIMT in TwinsUK that was not replicated in UKB. A sensitivity analysis including smoking status as a covariate was performed at the final stage of the analysis. The results remained unchanged, further supporting the robustness and reliability of our findings.

Using data from TwinsUK, the association between CWP and cfPWV was statistically significant in female participants aged older than 50 years, an observation that was also replicated in UKB data. In UKB, this association was also significant in younger male participants aged younger than 50 years, but not in older male participants.

Using the twin design, a common pathway model is suggested for CWP and carotid plaque with 95% of the latent factor variation attributed to genetic factors, but not to common environmental factors, and only 30% to unique environmental factors. The twin modelling suggests a latent factor underlies the CWP and cfPWV association, which is influenced by genetic factors (68%) and by common environmental factors (32%), suggesting a latent common pathway that explains the association. Thus, for the underlying factor associated with CWP and arterial stiffness, genetic factors and common environmental factors may play a significant role, while for CWP and the presence of plaque, genetic factors appear to play an important role. The genetic component of the common latent factor was found to account for as much as 65% of the variance explained by the genetics of CWP, 26% of cfPWV, and 34% of carotid plaque.

The 2-sample MR analyses showed a significant potential causal effect of CWP on the risk of coronary artery disease, but not the reverse. This result aligns with other MR studies conducted on multisite chronic pain, CVD, and coronary artery disease.^27,57^ The association between CWP and CVD has been well documented in recent research. Based on survival analysis of UKB, Rönnegård et al.^39^ showed that regardless of cardiovascular risk factors, CWP is an underestimated risk factor of CVD including myocardial infarction, stroke, heart failure, and cardiovascular mortality. The risk increment was stronger for heart failure, in line with a study derived from the Norwegian HUNT cohort.^52^ In this study of ample size, neither comorbidities nor demographic variables accounted for the association between heart failure and CWP.^52^ Several studies have reported excess mortality rates in people with CWP. A meta-analysis conducted by Macfarlane et al. using UKB 2017 data showed significant excess CVD-related mortality in people with CWP.^29^ In another meta-analysis, they found the association of increasing all-cause mortality among people diagnosed with fibromyalgia.^47^ A meta-analysis by Charalambos et all in 2020 showed that the aortic PWV and arterial stiffness increased the risk of CVD events and CVD all-cause mortality.^53^ Finally, studies in a number of different populations and settings have shown cfPWV, cIMT, and plaque-predict CVD events.^9,12,20,43,44,47,54^

Our study examined the association between CWP and cfPWV, cIMT and plaque. We found evidence suggesting an increase in cfPWV, cIMT, and plaque development in CWP. To date, only a few cross-sectional studies of small samples have revealed increasing cIMT and PWV in people with FM.^15,24,26^ Our study is among the first to focus on the role of CWP on arterial stiffness (cfPWV) and atherosclerosis (cIMT and plaque) in 2 large cohort studies and provides twin modelling as a method for disentangling environmental and genetic risk factors. This revealed that the link between carotid plaque and cfPWV with CWP may be explained by a putative latent component that is genetically based.

The progression of atherosclerosis is often attributed to inflammation, which also contributes to the stiffness of the arteries.^28,31^ A recent meta-analysis showed that increased levels of inflammatory cytokines (TNF-α, IL-6, IL-8, and IL-10) are reported in patients with fibromyalgia compared with healthy controls.^34^ These cytokines may have roles in CVD, atherosclerosis, and plaque formation.^17^ They could explain the role of latent factors and the latent common pathway between cfPWV and carotid plaque with CWP. Everson-Rose et al.^13^ (2021) studied 1399 postmenopausal female participants and showed that even after adjusting for CVD risk factors, there was increased PWV, greater carotid artery intima–media thickness, and decreased adiponectin levels. Higher carotid artery intimal thickness and slower fluctuations in pulse width were linked to elevated leptin levels, especially in female participants with diabetes or obesity. Another factor that may explain this latent pathway is depression, which has been shown to have a genetic correlation with CWP in the range of 0.3 to 0.6. While specific genetic correlation coefficients between depression and atherosclerosis or arterial stiffness are not well-documented, it is generally believed that there is some degree of shared genetic susceptibility. This shared susceptibility is thought to be mediated by mechanisms such as inflammation.

On the other hand, the CWP genome-wide association studies identified a genetic variant (rs10490825) in the gene ATPase secretory pathway Ca2+ transporting 1 (ATP2C1), which might explain the pathophysiology of calcified plaque formation. The review by Saba et al.^41^ discussed the role of calcium in atherosclerosis and the link between vascular calcification and plaque risk. In a previous study the TwinsUK data, Cecelja et al.^11^ demonstrated arterial stiffness is independent of noncalcified atherosclerosis but is associated with arterial calcification, which can be attributed to a shared hereditary effect on calcification and stiffness.

Age and sex have important impact on the observed association. A review study conducted recently to identify sex influences on plaque growth and atherosclerosis demonstrated that women experience a higher incidence of myocardial infarction at older age than men, although younger men manifest a greater atherosclerotic load and higher prevalence of ischaemic episodes up until the seventh decade.^36^ Compared with women, men acquire atherosclerosis around 10 years earlier in life, and one factor that may contribute to this is that men tend to have higher lipid profiles than women.^14,49^ Women have lower levels of LDL than men until they reach the age of 50 years, when LDL levels increased in women.^49^ This discrepancy may be explained by the protective effects of female hormones such as progesterone and oestrogen: menopause, accompanied by a dip in both these hormones, may increase a woman's risk of cardiovascular disease.^40,49^ Izumida et al.^19^ showed small dense low-density lipoprotein cholesterol (sdLDL-C) trends developed in men during young adulthood and subsequently dropped after the age of 50 years, but in women, the trends increased steadily until around at the age of 65 years. Płaczkowska et al.^35^ focused on sdLDL-C, which may be a better cardiovascular risk marker, and reported a significant concentration of sdLDL among people aged younger than 35 years, as well as discrepancies in sdLDL levels with concentrations of other lipids in both age groups. High sdLDL concentrations are associated with higher values of the standard biochemical risk factors of CVD.

There are several limitations to the study. First, in TwinsUK, CWP and arterial measures were not obtained at the same clinical visit, but we elected to include all those with CWP phenotyping within a 10-year period (2007-2016), and some twins had participated in the cardiovascular measurement visits. Compared with many prior studies, the sample size was adequate for women in middle age to investigate cfPWV, cIMT, and plaque association with CWP. Using a twin sample offers some advantages for twin modelling, but was not suitable for generalising the results through examination to men due to the low number of male participants in TwinsUK. Furthermore, most of the published publications that we used for our introduction and discussion part focused on the relation between CWP and CVD events, which is a broad term, but we could not find many publications focused on coronary artery disease, atherosclerosis, and arterial stiffness; more investigation is needed with considering these disease indicators.

Using UKB data, we replicated the finding of cfPWV association only in women older than 50 years suggesting that there may be an influence of menopause. In the UKB study, the cardio imaging visits for cIMT began at the second follow-up time point in 2014, and consequently, we found many individuals having both the cIMT measurements and reporting CWP.

## 5. Conclusions

The association between CWP and arterial stiffness and atheromatous plaque is due to the presence of a common pathway with an underlying latent genetic contribution of 68% and 90%, respectively. The genetic component of the common latent factor account for up to 65% of the variance explained by the genetics of CWP, 26% of cfPWV, and 34% of carotid plaque.

The epidemiological association between cfPWV and CWP was replicated in UKB in women aged older than 50 years. Overall, these results suggest that further investigation of the shared genetic mechanisms is warranted to understand the mechanisms by which CWP influences CVD leading to increased CVD-cause mortality rates in people with CWP.

## Conflict of interest statement

The authors have no relevant financial or nonfinancial interests to disclose. The authors have no conflicts of interest to declare that are relevant to the content of this article. All authors certify that they have no affiliations with or involvement in any organization or entity with any financial interest or nonfinancial interest in the subject matter or materials discussed in this manuscript. The authors have no financial or proprietary interests in any material discussed in this article.
